# Aesthetic Experience in the Acceptance of Wearable Technology for People With Dementia: Critical Interpretive Synthesis

**DOI:** 10.2196/72082

**Published:** 2025-11-06

**Authors:** Yixuan Wei, John Ratcliffe, Dag Aarsland, Wei Liu

**Affiliations:** 1 Department of Engineering, King's College London, Strand, London, WC2R 2LS, United Kingdom, 44 2078365454; 2 School of Healthcare, University of Leeds, Leeds, United Kingdom; 3 Institute of Psychiatry, Psychology and Neuroscience, King's College London, United Kingdom

**Keywords:** dementia care, wearable technology, aesthetics, health care design, assistive technology

## Abstract

**Background:**

The prevalence of dementia has led to a growing interest in wearable technologies to assist dementia care. Despite their potential, these technologies face low adoption rates, often attributed to poor aesthetic design and insufficient consideration of user experience.

**Objective:**

This study aims to (1) explore how the aesthetic design of wearable devices relates to their adoption and user experience in dementia care and (2) critically examine the ways in which aesthetic elements shape people with dementia’s perceptions of acceptability and inform future design considerations.

**Methods:**

A critical interpretive synthesis with a systematic search was conducted across 2 databases, namely Web of Science and Scopus on August 22, 2024. Studies were included if they reported on the current use of wearable technologies in dementia care or provided value in qualitative studies addressing attitudes from people with dementia and their caregivers toward the wearable product. Two authors independently screened the abstracts and full texts to extract data, and additional studies were included from web searches, owing to their conceptual contributions to offering insights into the emergence of wearable technology, including the factors driving its commercial value and appraisal.

**Results:**

A total of 63 studies were included in this study. Findings suggest that aesthetically considered designs are preferred by users when concerning their acceptance toward wearable devices, particularly when devices symbolize empowerment and support personal engagement. The objects that evoke comfort, emotional connection, and personal meaning are more likely to be accepted by people with dementia. Improved aesthetics may also support caregivers through more consistent and effective data collection.

**Conclusions:**

This study uncovers a significant gap in the aesthetic design of wearable technologies for dementia care, limiting user acceptance and emotional engagement. By synthesizing key themes focusing on the interaction between user and product, this review proposes a conceptual framework for dementia care, emphasizing the importance of aesthetics in enabling more meaningful, inclusive, and human-centered design.

## Introduction

### Background

Dementia is an incurable disease that causes brain damage from various conditions, contributing to disability and severe dependency, particularly in older adults [1]. In 2023, over 37 million people were diagnosed with dementia, with a projection for the number to rise to 10 million annually, reaching over 300 million by 2050 [1]. As dementia diagnoses increase, greater strain is placed on caregivers and health care systems, affecting the mental and physical well-being of those involved [2]. To date, smart wearables equipped with sensors to monitor daily health data or provide support are considered promising tools to ease these burdens [3,4]. Although a growing number of studies have shown an interest in testing the feasibility of wearable technologies in dementia care, there remains a low adoption rate among patients in accepting wearable devices [5]. While aesthetics is a critical factor that often directly influences the adoption of devices [6], its role in wearable technology design, particularly in dementia care, remains underexplored. This study addresses this gap by investigating how aesthetic factors contribute to user engagement and experience in an exploratory context in dementia care.

### Dementia

Dementia encompasses a range of progressive symptoms characterized by the decline of cognitive and functional abilities, often associated with neuronal loss in the hippocampus and cerebral cortex [7]. Common forms include Alzheimer disease and vascular dementia, both of which significantly impact daily functioning [8]. As the condition progresses, individuals often experience memory loss, impaired communication, and behavioral changes, which are frequently accompanied by behavioral and psychological symptoms, such as the emergence of agitation, aggression, and anxiety [9]. While research on wearable technologies’ application in dementia care has shown significant potential in early-stage disease detection [10,11], GPS location tracking [12], and daily activity monitoring [13-15], most have prioritized feasibility over design considerations. As a result, issues of stigma directed toward people with dementia have persisted, potentially influencing their willingness and ability to use these products [16,17]. Aesthetics play a crucial factor in users’ acceptance and adoption of these devices, encouraging individuals to try and adopt devices intended for continuous health monitoring and long-term well-being [14].

### Aesthetic Experience in Dementia Care

As emerging technologies, such as virtual reality and wearable devices, become increasingly integrated into health care and daily life, there is growing recognition that their success depends not only on functional performance but also on the quality of user experience [18,19]. Aesthetic experience, thus, can be addressed to help understand how individuals perceive, engage with, and emotionally respond to a product [20].

Aesthetics contributes to a sense of delight and appreciation when engaging with a product, often through sensations of beauty, amusement, and pleasure derived from sensory experiences, such as touch, sight, or sound [20]. The impressions left by aesthetics are deeply tied to how individuals physically interact with objects in their environment. Beyond the level of appeal from the appearance, design often carries symbolic meaning, where objects also evoke the development of cultural and personal associations. This symbolic layer of aesthetics reflects how users interpret and internalize a product’s presence, which can influence not only their comfort and engagement but also their sense of identity and connection. Although dementia progressively impairs memory and the processing of selfhood, it is argued that the selfhood and one’s aesthetic awareness can persist despite cognitive decline, such as how one dresses or behaves in a social context [21]. While stigma has been identified as a significant concern for people with dementia, who may face discrimination and ageism, the preservation of self-image, thus, plays a crucial role in shaping how individuals perceive their appearance and social standing [22]. To this extent, understanding both the physical engagement and symbolic meaning in aesthetics from the interaction between people with dementia and their surroundings offers valuable insights for designing and conceptualizing technologies that are not only usable but also emotionally and socially resonant.

### Research Aim

This research aims to critically investigate (1) how wearable technologies are currently applied in dementia care and (2) how these technologies are experienced in terms of aesthetics and long-term engagement from the perspectives of people with dementia. In response to the limited theoretical development in this area, this review adopts a critical interpretive synthesis (CIS) approach to engage with the existing literature. This method enables a critical exploration of whether and how aesthetic values are considered by people with dementia through their interactions with the devices. By emphasizing the role of aesthetics in user acceptance, this study seeks to inform the design of wearable technologies that prioritize comfort, beauty, and personal identity, thereby supporting sustained use, enhancing quality of life, and increasing the impact of assistive technologies in dementia care.

## Methods

### Design

This review used CIS to critically examine existing literature on the application of wearable technology in dementia care, focusing on aesthetic design. CIS is instrumental in generating innovative theories by synthesizing evidence for deeper insights into the topic. It merges systematic review methods with qualitative analysis to examine both empirical and nonempirical studies [23]. A flow diagram is generated to assess literature eligibility and illustrate the clarity of the data selection process, with 2 reviewers involved to ensure diverse perspectives. This approach enabled the identification of key insights and future research directions, particularly on inclusive wearable design and its impact on patient adoption and data collection in dementia care.

### Search Strategy

This review collected studies from 2 well-grounded databases, namely Scopus and Web of Science. The search was conducted on August 22, 2024. An example of the search term is (jewel* OR wear* OR accessory* OR fashion* OR craft*) AND dementia* (TOPIC) on Web of Science, and the language is limited to English (LANGUAGE). The year limit and journal types have not been set in the literature search to allow flexibility to offer a broad view of theories and practices of wearable devices and their application in dementia. Altogether, 3 additional studies [24-26] were added to the inclusion list of the reviewed studies from a web search, owing to their conceptual contributions to offering insights into the emergence of wearable technology, including the factors driving its commercial value and appraisal [24-26].

### Study Selection

The inclusion criteria of studies are considered relevant based on the following: (1) studies relevant to technology that physically touches and interacts with people with dementia, (2) a feasibility study on applied wearable technology, and (3) interviews or discussions toward the existing or preferred wearables from the perspectives of people with dementia. Studies in different disciplines and perspectives (eg, discussion of aesthetics for assistive devices, the commercial market of wearable technology, and product design framework) are considered helpful and offer insights into data analysis. The exclusion of studies involving irrelevant topics or unclear presentations of the wearable prototype.

### Quality Appraisal

This study adopted a combination of quality criteria to guide the literature review process. The initial selection of literature from 2 databases followed a structured and transparent filtration procedure, documented through a PRISMA (Preferred Reporting Items for Systematic Reviews and Meta-Analyses)-style flowchart. Following the principles of CIS, additional conceptually significant sources were purposefully sampled beyond the initial search, including gray literature, such as a website of a product and materials identified via Google Scholar. These were included for their value in illuminating the symbolic positioning and public perception of wearable technologies in contemporary culture [23,27]. Two reviewers have been assigned to conduct a full-text review independently to determine the final inclusion of studies in the review.

### Data Extraction

Data extraction was conducted by the lead author using Microsoft Excel, designed to systematically capture information relevant to the research aims. Extracted details included study authors, domain, design, country, body location of wearable application, types or brands of wearable technologies, and whether the study addressed practical feasibility, aesthetic value, or both.

**Figure 1.**
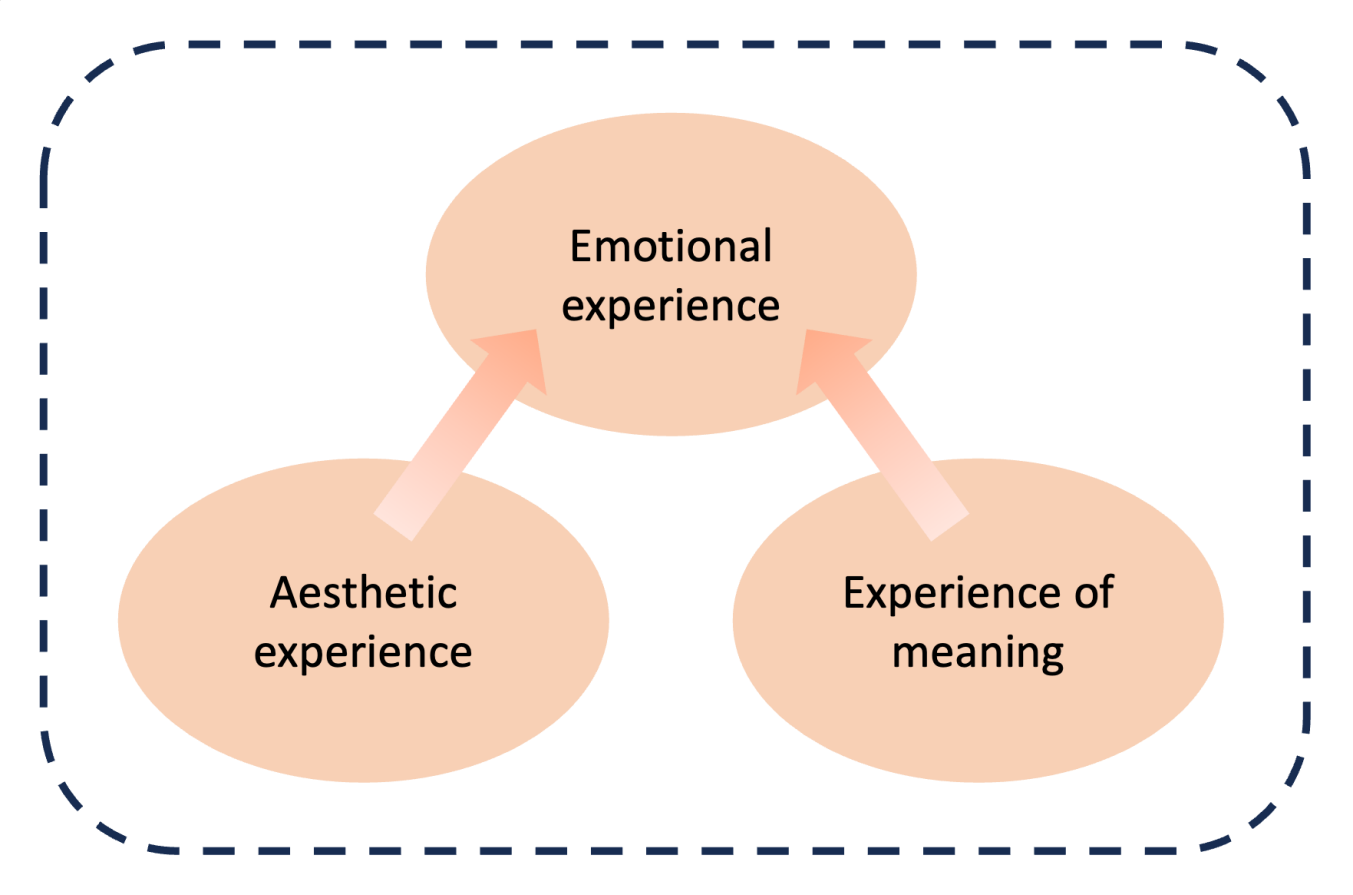
Product experience framework (adapted from Desmet and Hekkert [20], which was originally based on the earlier work of Hekkert [28]). Published under Creative Commons Attribution 4.0 License [CC BY 4.0] [29].

The inclusion of aesthetic value was guided by Desmet and Hekkert’s [20] conceptual framework, selected for its relevance in examining how aesthetics shapes user-product interaction (Figure 1). The term product was interpreted broadly to include artifacts with tangible features (eg, shape, texture, and color) designed for user engagement.

### Data Synthesis

Further coding of studies identified as engaging with the aesthetic value of the product was carried out by 2 reviewers using the 3 themes. The framework served as a sensitizing tool, providing a conceptual lens while allowing interpretive flexibility in line with CIS. For instance, discussions of device appearance (eg, preferred color and weight) informed the aesthetic experience dimension, while references to personal significance or identity informed the experience of meaning dimension. Given the interrelated nature of the themes, studies were allowed to span multiple categories where appropriate. Each reviewer conducted independent coding, followed by a collaborative discussion to resolve discrepancies and ensure coherence in categorizing the themes.

Findings were presented through a combination of tables, visuals, and narrative synthesis. One table summarized all included studies, while a second focused specifically on the distribution of aesthetic themes. Thematic analysis, which was initially guided by the referenced framework, was then explored under the dementia care context. Insights gained from this process informed the development of a new conceptual framework to better capture the role of aesthetic values in the design and acceptance of wearable technologies for people with dementia.

## Results

### Search Results and Analysis of the Wearables

**Figure 2.**
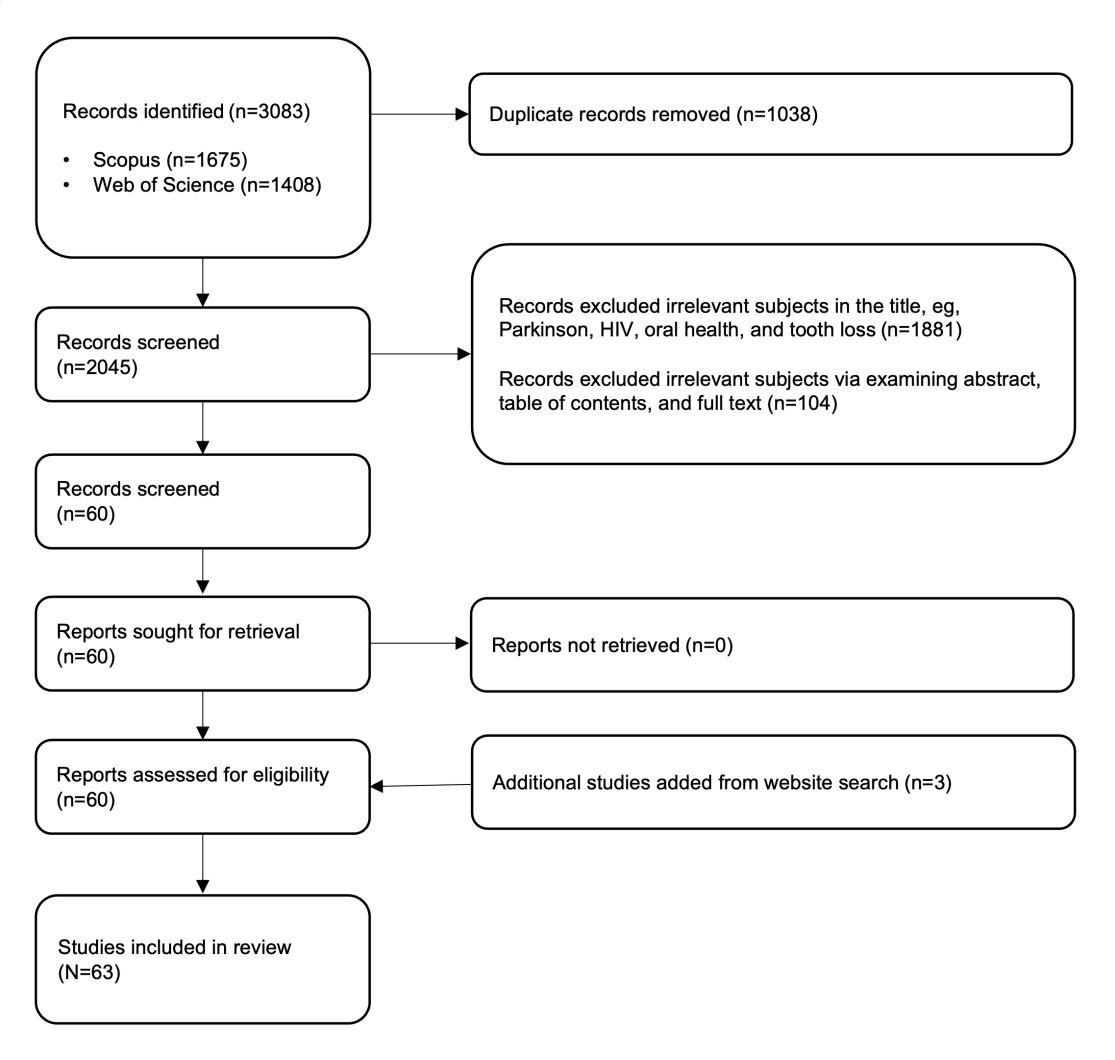
Flow diagram of reviewed work.

The initial search returned 3083 results (1675 from Scopus and 1408 from Web of Science). Following the application of exclusion criteria, 60 studies [4,5,11-15,18,30-81] were included. An additional 3 studies [24-26] were purposefully added for their conceptual relevance to the aesthetic and commercial dimensions of smart wearable devices, bringing the total to 63 studies [4,5,11-15,18,24-26,30-81] (Figure 2). The complete list is presented in Table 1.

**Table 1.** Reviewed studies distribution chart.

Literature reference	Study domain	Study design	Country	Body location	Wearable technology applied	Practical application	Aesthetics value
Apple [26]	N/A^a^	Website or Gray literature	United States	Wrist	Apple Watch Series 6		✓
Alam et al [14]	Dementia, agitation	Iterative study	United States	Wrist	The Pebble Watch	✓	
Alam et al [15]	Dementia	Transdisciplinary study	United States	Wrist	The Pebble Watch	✓	
Amato et al [30]	Alzheimer	Mixed methods	Italy	Wrist	Empatica E4, the Eclipse	✓	✓
Anwar et al [11]	Vascular dementia	Simulation and experimental design	United Kingdom	Eye	smart Radio Frequency (RF) glasses (proposed)	✓	
Appel et al [31]	Dementia, BPSD^b^	Pilot Study	Canada	Head	Samsung Gear VR^c^ HMD^d^ with Sennheiser HD 221 headphones	✓	✓
Mc Ardle et al [32]	Alzheimer, mild cognitive impairment	Feasibility study	United Kingdom	Wrist	AX3 Axivity	✓	
Mc Ardle et al [33]	Alzheimer	Pilot study	United Kingdom	Waist	AX3 Axivity	✓	✓
Badawi et al [34]	Dementia, BPSD	Mixed methods	Canada	Wrist	Empatica E4	✓	
Boletsis et al [35]	Dementia	Qualitative feasibility study	Norway	Wrist	Basis B1	✓	
Chan et al [36]	Dementia	Feasibility and validity	Australia	Wrist	AX3 Axivity	✓	
Cheung et al [37]	Dementia	Qualitative study	United Kingdom	Head	Meta Quest 2	✓	✓
Cheung et al [38]	Dementia, agitation	Scoping review	China	Wrist	N/A	✓	✓
Choi and Kim [24]	N/A	N/A	South Korea	Wrist	N/A		✓
Chung et al [39]	Dementia	Dyadic case study	United States	Wrist	Garmin Vivoactive HR^e^	✓	✓
Cullen et al [4]	Dementia	Systematic review	United Kingdom	Wrist	N/A	✓	
Davidoff et al [40]	Dementia, agitation	Multimodal system design	Belgium	Wrist	Chill+	✓	
Davis and Sikorskii [41]	Alzheimer	Feasibility Study	United States	Head	Eye tracking glasses with joystick	✓	✓
Farina et al [42]	Dementia	Feasibility study	United Kingdom	Wrist	GENEactive Original	✓	✓
Favela et al [43]	BPSD	Mixed methods	Mexico	Wrist	Fitbit Charge 2 HR	✓	✓
Felber et al [44]	Dementia	Qualitative study	Switzerland	N/A	N/A		✓
Guu et al [45]	Dementia, Alzheimer	Review study	United Kingdom	Wrist	N/A	✓	✓
Guu et al [46]	Dementia	Feasibility study	United Kingdom	Wrist	GENEactive	✓	✓
Harper and Ghali [47]	Agitation and aggression, BPSD	Systematic review	United Kingdom	Wrist	Empatica E4, The Philips Discrete Tension Indicator 2 (DTI-2), The Pebble watch, The Eclipse	✓	
Hassan et al [48]	Dementia	Qualitative Study	United Kingdom	Wrist	Fitbit Charge HR		✓
Imtiaz et al [49]	Dementia, BPSD	Design experiment	United States	Wrist	Empatica E4	✓	
Iwata et al [50]	Dementia	Control study	Japan	Wrist	Omron wristband	✓	
Kamil et al [51]	Alzheimer	Feasibility study	United States	Waist	Opal	✓	
Kikhia et al [52]	Dementia, BPSD	Feasibility study	Sweden	Wrist	The Philips DTI-2	✓	
Kikhia et al [53]	Dementia, BPSD	Feasibility study	Sweden	Wrist	The Philips DTI-2	✓	
Klimek et al [54]	Dementia, BPSD	Scoping review	The Netherlands	N/A	N/A	✓	✓
Kuzmik et al [55]	Dementia	Feasibility study	United States	Wrist	MotionWatch 8	✓	
Larnyo et al [56]	Dementia	Cross-sectional survey	China	N/A	N/A		✓
Lastovicka-Medin [57]	Dementia	Research design	Montenegro	N/A	e-Textile (self-developed)	✓	✓
Lee et al [58]	Dementia	Qualitative Study	South Korea	Head	VR Device with EEG^f^ Headset	✓	
Li et al [59]	Alzheimer	Research design	China	N/A	e-Textile (self-developed)	✓	✓
Mahoney and Mahoney [60]	Alzheimer	Interviews	United States	N/A	N/A		✓
Marcén et al [61]	Dementia, agitation	Feasibility study	Spain	Wrist	GENEactive Original	✓	
Megges et al [18]	Dementia	Comparative study	Germany	Wrist	HIMATIC GPS Uhr Alpha; ReSOS-2—Die Notfalluhr;	✓	✓
Mohamedali and Matoorian [62]	Dementia	Research study	United Kingdom	N/A	N/A		✓
Mohammed and Mohammed [63]	Dementia	Design research	United States	N/A	N/A	✓	✓
Murphy et al [64]	Dementia	Cross-sectional study	United Kingdom	Arm	Sensewear Armband	✓	
Musaeus et al [65]	Alzheimer	Feasibility study	Denmark	Ear	Ear EEG (self-developed)	✓	✓
Nieroda et al [25]	N/A	Qualitative study	United Kingdom	Wrist	N/A		✓
Oh and Gross [66]	Dementia	Design research	United States	Feet	Awareable steps (self-developed)	✓	✓
O’Sullivan et al [67]	Dementia	Mixed methods	The Netherlands	Wrist	Fitbit Charge 3	✓	✓
Peeters et al [68]	Dementia, BPSD	Qualitative study	The Netherlands	Wrist	Empatica E4	✓	✓
Rings et al [69]	Dementia	Qualitative Study	Germany	Head	VR Device with crafted Camera	✓	✓
Schneider and Henneberger [12]	Dementia	N/A	Austria	Wrist	Laipac S911	✓	✓
Silva et al [70]	Alzheimer	Experimental study	Portugal	Chest	SenseCam	✓	
Spasojevic et al [13]	Agitation and aggression, BPSD	Pilot study	Canada	Wrist	Empatica E4	✓	
Staab et al [71]	Dementia	Experimental study	Germany	Wrist	Apple Watch Series 6	✓	
Stavropoulos et al [72]	Alzheimer	Public involvement activities	Greece	Wrist	N/A	✓	✓
Stavropoulos et al [73]	Dementia	Design study	Greece	Wrist	The Philips DTI-2, UP24	✓	
Tervonen et al [74]	Dementia	Design study	Finland	Neck	Self-developed sensor	✓	
Thorpe et al [75]	Dementia	Feasibility study	Denmark	Wrist	Sony SmartWatch 3	✓	
Thorpe et al [76]	Dementia	User-centered approach	Denmark	Wrist	Sony SmartWatch 3	✓	
Thorpe et al [77]	Dementia	Mobility measurement module	Denmark	Wrist	Sony SmartWatch 3	✓	
Treadaway and Kenning [78]	Dementia	Design research	United Kingdom	N/A	e-Textile (self-developed)	✓	✓
Wallace et al [79]	Dementia	Cocreative, design-led	United Kingdom	N/A	Self-developed jewelry		✓
Woodberry et al [5]	Alzheimer	Participatory study	United Kingdom	Neck	SenseCam	✓	
Yokoi et al [80]	Dementia, BPSD	Exploratory study	Japan	Finger	Ring		✓
Zeng et al [81]	Dementia	End-to-end deep learning method	Japan	Head	EEG cap (self-developed)	✓	✓

a N/A: not applicable.

b BPSD: behavioral and psychological symptoms of dementia.

c VR: virtual reality.

d HMD: head-mounted display.

e HR: heart rate.

f EEG: electroencephalogram.

A total of 63 studies [4,5,11-15,18,24-26,30-81] met the eligibility criteria, showcasing a wide variety of wearable devices used on different parts of the body from head to toe. The wrist was the most frequently targeted area, with 37 studies [4,12-15,18,24-26,30,32,34-36,38-40,42,43,45-50,52,53,55,61,67,68,71-73,75-77] focusing on wrist-worn devices that often feature display screens and 3-axis accelerometers, mainly used to record motion data [47]. Among these products, the Empatica E4 was the most tested device for monitoring physiological signals like heart rate variability and electrodermal activity [47], especially for everyday activity tracking and agitation detection [13,30,34,47,49,68]. The Philips Discrete Tension Indicator has also been appraised in dementia care to monitor physical indicators and stress levels [47,52,53,73]. Likewise, the AX3 Axivity device has been studied for its feasibility in dementia care, especially for gait analysis [32,33,36]. Beyond wrist devices, an armband with a triaccelerometer has been used to monitor daily nutrition intake in people with dementia [64]. Waist-worn sensors have been tested for detecting wandering behaviors [51]. Body-worn SenseCams, worn on the neck or chest, show promise for memory stimulation in patients with Alzheimer disease [5,71]. In addition, a neck-worn laser device aids navigation [74] while footwear sensors have been explored for alerting and monitoring purposes [66]. For headwear, virtual reality (VR) devices are seen as merging innovative technologies that often offer rich sensory engagement, promoting active physical interaction for people with dementia, such as reaching out with hands or feet and rotating the body while exploring an immersive 3D environment [31,37,41,58,69].

Around 35 studies [12,18,24-26,30-32,37-39,41-46,48,54,56,57,59,60,62,63,65-69,72,78-81] were assessed for alignment with 3 core themes of the framework, namely aesthetic experience, experience of meaning, and emotional experience [20]. These themes were developed to examine how wearables engage users beyond functionality, encompassing aesthetic involvement, self-image, and emotional connection. They were informed by theoretical foundations in product experience and patterns identified in the extracted data.

A detailed breakdown of the studies that addressed esthetic considerations is presented in Table 2, with a clear distribution presented under three referenced thematic categories.

**Table 2.** Distribution of studies related to aesthetic value.

Literature reference	Applied product	Aesthetics experience	Experience of meaning	Emotional experience
Schneider and Henneberger [12]	Laipac S911BL Xexun TK202 Xexun TK203 Xexun TK102-2 CRT 19N CY 2130 Teletonika GH 3000	Recommended size: 50×50×20 mm, ≤100 g. Panic button essential. Bracelet or watch design preferred	—^a^	—
Megges et al [18]	HIMATIC GPS Uhr Alpha, ReSOS-2	Preferred: button-based, minimal design.	Design perceived as stigmatizing.	—
Choi and Kim [24]	Smartwatch	—	Smartwatches are seen as innovative, luxury fashion items.	—
Nieroda et al [25]	Wearable devices	—	Wearables are viewed as hybrid products: smart and innovative.	—
Apple [26]	Apple Watch Series 6	—	Apple Watch linked to a high-tech, healthy lifestyle. Fashion ties reinforce the stylish image.	—
Amato et al [30]	Empatica E4	Tactile features aid engagement. E4 is seen as thick, tight, and uncomfortable.	—	—
Appel et al [31]	Samsung Gear VR^b^	Device linked to heaviness, nausea, and discomfort Rich sensory engagement with movement aided by sound cues.	—	Reported relaxation, happiness, stress relief, and benefits linked to brief wear rather than extended use.
Mc Ardle et al [32]	Axivity AX3	The device is comfortable and easy to wear.	—	—
Cheung et al [37]	Meta Quest 2	Immersive art or crafting experience with colorful tools.	—	Enabled expression and engagement with personalized, meaningful aspects.
Cheung et al [38]	Wrist-worn accelerometer devices	The issue of comfort has been addressed.	—	—
Chung et al [39]	Garmin Vivoactive HR^c^	Preferred watch-like monitor with on-screen display.	—	—
Davis and Sikorskii [41]	VR Glasses or Goggles Applied Science Laboratory	Clear glasses with joysticks supported active body engagement.	—	—
Farina et al [42]	GeneActiv	Comfort, size, weight, usability, safety, security, and durability valued.	Watch-like design seen as nondistinctive, causing issues for existing watch wearers.	—
Favela et al [43]	Fitbit Charge 2 Fitbit Alta	—	Preferred discreet design. Lacked self-image value.	—
Felber et al [44]	Wearable devices	Existing wearables seen as unappealing and clunky.	—	Wearables caused feelings of incapacity. Complex functions discouraged users.
Guu et al [45]	Wearable devices	—	—	Older adults underrepresented in wearables. Ageism needs addressing.
Guu et al [46]	GeneActiv	Suggested: a more feminine device with a time display.	Watch-like design confused participants already wearing a watch.	—
Hassan et al [48]	Axivity AX3 Fitbit Charge HR; MOTO 360 Garmin Vivogit 2 Misfit Speedo Shine Withings Activite Pop Withings Pulse Ox	Water resistance and ease of use were identified as critical factors.	The device may draw unwanted attention.	The device may cause the wearer to feel incapable.
Klimek et al [54]	Empatica E4	Watch-like design recommended.	—	—
Larnyo et al [56]	Wearable devices	Discomfort was reported as an issue by wearers.	—	Reported issues: frustration, confusion, embarrassment, and anxiety.
Lastovicka-Medin [57]	Self-developed sensored textile	e-Textiles responded to touch, fostering engagement and connecting past and future knowledge.	—	—
Li et al [59]	Self-developed sensored textile	Soft, colorful textiles promoted communication and tactile engagement.	—	—
Mahoney and Mahoney [60]	Wearable devices	Recommended: waterproof, jewelry-like, easy to clean, and hypoallergenic.	Products were perceived as institutional and intrusive.	The products were perceived as diminishing wearer dignity.
Mohamedali and Matoorian [62]	Wearable devices	Preferred: comfort and varied straps or mounts (colors, sizes, and materials).	Preferred: less obtrusive, discreet design.	Wearable presentation undermines users’ sense of capability.
Mohammed and Mohammed [63]	Wearable devices	A “watch-like” design is preferred.	—	—
Musaeus et al [65]	Self-proposed ear EEG^d^	Recommended: Smaller amplifier, improved textures. Mild ear discomfort reported.	Wearing the device may draw unwanted attention.	Wearables may reveal vulnerability, discouraging use.
Oh and Gross [66]	Self-developed shoes	Soft leathers were incorporated for comfort and visual appeal.	—	—
O’Sullivan et al [67]	Fitbit Charge 3	—	Participants may forget or avoid the watch-like device. Adds little user value.	—
Peeters et al [68]	Empatica E4	Preferred: water-resistant, easy-to-use, comfortable, personalized (colors or jewelry). E4 caused tightness and discomfort.	—	Device’s appearance raised fear of unwanted public attention.
Rings et al [69]	VR Device with Crafted Camera	Wearers performed active movements, including body exercises.	—	Game-based designs offered immersive experiences, supporting personal stories and memories.
Stavropoulos et al [72]	Wearable devices	Preferred: water-resistant, soft materials, and varied colors.	—	—
Treadaway and Kenning [78]	Self-developed textile	Rich-textured, soft design evoked attachment and affection.	—	—
Wallace et al [79]	Self-developed jewelry	Self-made jewelry with personalized forms and varied materials.	—	Personalized jewelry fostered emotional attachment.
Yokoi et al [80]	Jewelry	Aesthetically designed stone rings.	—	Stone rings enhanced confidence, beauty, and dignity.
Zeng et al [81]	Self-developed head EEG	Textile EEG cap designed for stable skin contact and comfort.	—	—

a Not applicable.

b VR: virtual reality.

c HR: heart rate.

d EEG: electroencephalogram.

### Aesthetic Experience

Aesthetic experiences engage the senses and shape user interactions with the product [20]. On this level, the aesthetic connections start by giving pleasure to the wearer’s sensual feelings, whether it is an attractive appearance or an amusing sound. In the reviewed studies, wrist-worn wearables, mostly designed as smartwatches with screens and strap bands, such as Basis B1, Sony SmartWatch 3, Apple Watch Series 6, and Pebble Watch, are the most commonly used devices in dementia care [35,40,47,50,71,75-77], although some have watch-like forms but without screens [55,61]. The aesthetics of having the wrist-worn devices with a “watch-like” design are also recommended by several studies [12,39,54,63]. In the considerations of tactile senses, materials with waterproof quality have been discussed as necessary to cope with different environments [48,60,72]. Comfort is another critical factor that greatly influences users’ opinions toward assistive technology while the product is intimately engaging with the wearer’s body [38,56]. It is also suggested that materials should be hypoallergenic and washable to ensure safety and comfort [60,72]. However, overly secure fastenings, while beneficial for data consistency, can increase anxiety or agitation, emphasizing the need for user-friendly designs [68]. Regarding the wearable’s weight and size, it is mentioned that the artifact should weigh under 100 g and not exceed dimensions of 50x50x20 mm [12], with miniaturized designs preferred for discretion [18,60,65]. Tight-fitting devices, such as the Empatica E4, have been criticized for causing discomfort and skin marks [68]. Concerning the personalization of wearable design aesthetics, feminine appearance [46], different colors and various material options are preferred [12,44,62,68,72].

In the development of VR experiences, comfort has also been a concern, with reports of simulation sickness and heaviness, including mild nausea associated with wearing headsets [31]. Despite these issues, to further enhance sensory immersion, studies have combined VR headsets with joysticks, tangible objects, and earphones, allowing users to experience not only visual pleasures but also tactile and auditory stimuli, fostering a more embodied and present experience [31,41,69].

Soft materials and objects with textured surfaces are highly valued in the responses of individuals with dementia [57,59,66,78,81]. While footwear naturally becomes part of people’s daily outdoor routines, devices like smart shoes made from soft brown leather demonstrate how fashionable designs can seamlessly blend into everyday life while meeting functional needs [66]. For textiles, a study has begun designing a hat using soft materials to cover EEG sensors for emotional detection [81]. A co-design study’s examination revealed that interacting with tactile objects enhances emotional well-being and social connections for those affected [78]. Personal reminiscence can also be triggered through the crafting process with textiles [57]. It is also found that a handkerchief with varied colors, designed to assist pain communication for individuals with Alzheimer disease, has been decorated and accepted [59].

### Experience of Meaning

A meaningful experience involves a user’s interaction with a product that engages deeper cognitive processes, such as interpretation, memory recall, and the formation of personal associations. Symbolism in design influences how users envision their lives with an object, extending its impact beyond simple sensory pleasure. For example, luxury homeware may symbolize comfort and a high standard of living [20]. Similarly, wrist-worn devices, especially those styled like watches, often represent innovation and intelligence by combining functions, such as time display with health tracking [24,25]. Unlike traditional devices, smartwatches like the Apple Watch are more closely integrated with the body. The design, featuring customizable bands and collaborations with brands like Hermès, has strengthened their dual role as both technical tools and luxury fashion accessories [26]. In dementia care, however, this symbolic link to health and lifestyle may pose new challenges.

In the context of dementia, individuals often prefer discreet designs with smaller displays, mainly to avoid unwanted attention and public anxiety [43]. Instead of conveying wellness or a high quality of life as intended by watch-like wearable designs, such devices can cause embarrassment and discomfort due to their visibility in public settings [60,62]. This reveals a key conflict in the symbolic meaning of smart technologies. While they are often linked to innovation and well-being, for those already dealing with health issues, these devices can express fear and a wish to hide. A visible device may be seen by others as a sign of incapacity, making wearers feel vulnerable or stigmatized [65]. The perception that these devices give an “institutional look” to people with dementia has reinforced negative symbolism and may reduce the user’s sense of dignity and autonomy [60]. Moreover, for many who already own watches with personal significance, the addition of a second watch focused solely on health monitoring can cause confusion and resistance, especially when users are unsure why they are wearing 2 watches [42,46,67].

### Emotional Experience

Emotional experience arises from an individual’s interpretation of a product rather than from the product itself [20]. In dementia care, preferences for discreet presentation reflect concerns that negative emotional responses to smart wearables are less about the devices themselves and more about what they represent, including monitoring, dependency, and declining health. Wearable technologies have thus been associated with symbols of vulnerability and constant surveillance [65]. As quality of life in later years is closely linked to autonomy and social connectedness, the current design of wearable devices can act as a social barrier by unintentionally signaling a need for assistance and thereby diminishing confidence and willingness to engage socially [31,44,45,48,56,60,62,65]. This underscores the importance of exploring how potential emotionally threatening associations could be improved or transformed through more positive human-product interactions by examining cases where people with dementia experience pleasant emotional responses to wearable objects.

In studies reporting positive emotional responses among people with dementia, both aesthetic experience and personal meaning appear to play crucial roles in shaping the likelihood of wearable adoption. From an aesthetic perspective, recent evidence highlights a preference for wearables designed as fashion accessories, particularly jewelry-like forms, which are perceived as more acceptable than watch-like designs [60,68]. While emotions play a key role in forming attachment to products, studies have shown that women with dementia displayed increased grace and self-awareness when wearing gemstone rings provided during the study [80]. Although based on a small sample, participants expressed positive feelings by using appreciative words, such as “fancy” and “beautiful,” highlighting the empowering potential of aesthetics through jewelry.

In relation to the experience of meaning, products that incorporate personalized stories or narratives have been shown to elicit positive emotional responses. For example, a co-design project developed personalized jewelry pieces for a woman with dementia, who described them as emotionally meaningful and deeply personal. This case highlights the importance of aesthetic and symbolic connections in the design of wearable objects [79]. As a head-mounted technology, VR has shown promise in providing immersive experiences for people with dementia, supporting momentary engagement and self-expression through interaction with virtual reality environments [37,69]. Studies have enabled users to personalize scenes, such as cocreating meaningful spaces from physical to virtual reality presentation [37] or having the flexibility to take photos that one likes under a built environment [69].

While centered on fostering positive experiences for people with dementia through the aesthetic dimension, these cases highlight the importance of wearable technologies that are both visually appealing and capable of personalization. Such design approaches may encourage positive emotional connections and facilitate the development of long-term attachment to the product within dementia care.

## Discussion

### Principal Findings

This study underscores the crucial role of aesthetics in influencing how people with dementia adopt wearable devices. Key factors include the sensory experience of the product, symbolic associations that shape perceptions of empowerment versus stigma, and the significance of personal narratives in fostering emotional connections with the device. All of these should be carefully considered in the design for dementia and inclusive care.

### New Conceptual Framework for Dementia

**Figure 3.**
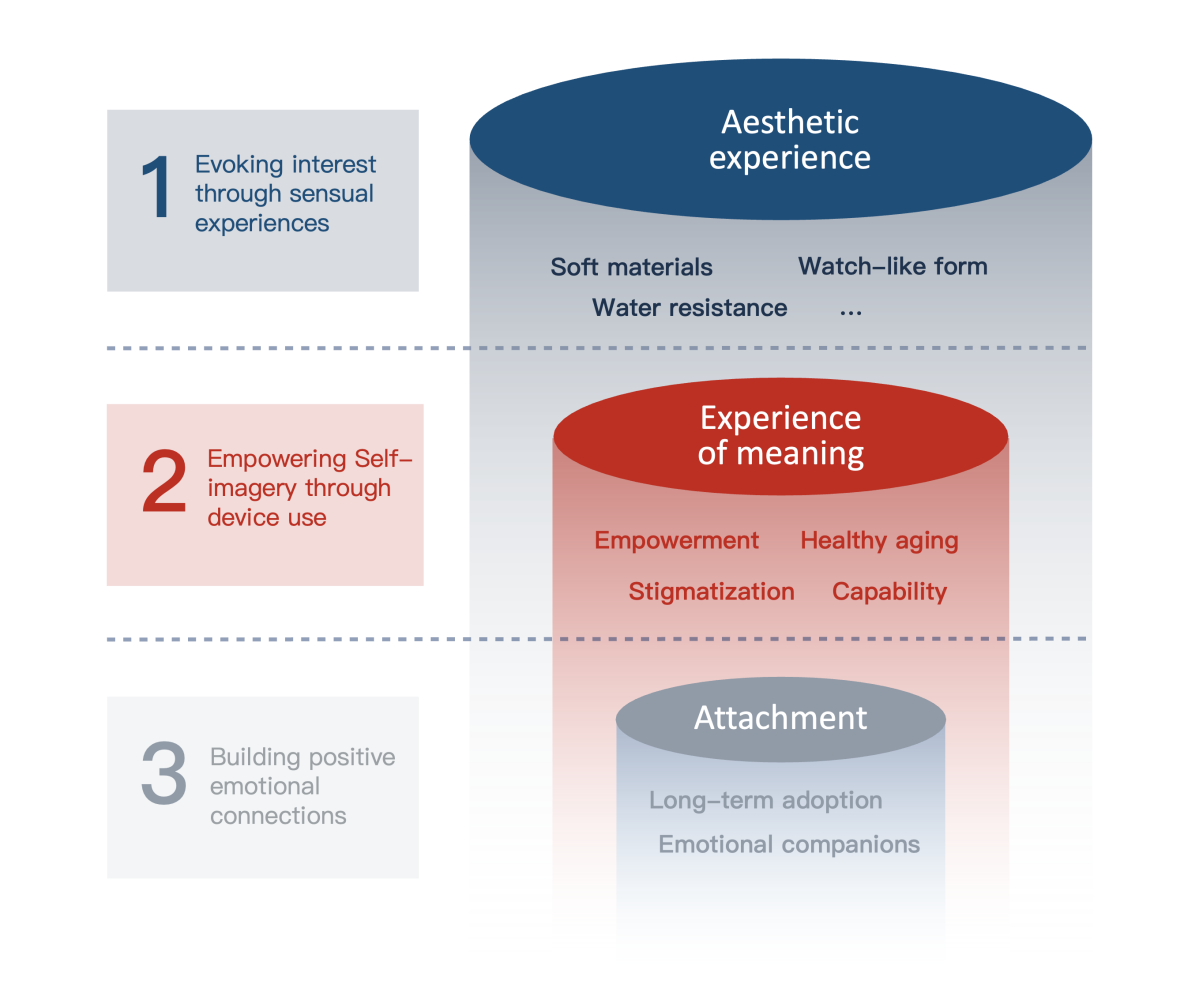
User-wearable product framework for dementia care.

Building on an existing framework that links aesthetic, emotional, and meaningful experiences in human-product interaction [20], this review introduces a new conceptual model (Figure 3) based on the perspectives of people with dementia toward current wearable technologies and studies exploring the aesthetics of wearable objects in dementia care. While the original framework highlights the potential interlinking of the 3 elements, our findings indicate that they need to be directly interconnected in studying and designing for a better experience between people with dementia and wearable technologies. This begins with generating interest through aesthetic appeal and addressing concerns about uncomfortable or unappealing designs. It then moves to shaping a positive self-image that fosters empowerment rather than incapacity. Finally, it culminates in building lasting emotional connections with the device, encouraging continued use and supporting both physical and mental well-being. To encourage acceptance, long-term use, and emotional engagement, future design of wearable devices should consider both aesthetic appeal and the personal significance they may hold for individuals with dementia.

As illustrated in Figure 3, the interaction between people with dementia and wearable devices is conceptualized in 3 key stages. The first stage involves an initial attraction driven by the product’s sensory appeal, where the aesthetic experience is primarily based on sensual appreciation. This early interest encourages deeper engagement in the second stage, where individuals begin to form a personal connection with the device. At this point, the product should empower users by supporting health, offering comfort, and providing clear benefits without reinforcing stigma or perceptions of incapability. Together, these first 2 stages create space for personal interpretation and self-expression, allowing individuals to build meaningful narratives of their own with the device and experience emotional resonance. Ideally, this process culminates in a third stage of attachment, in which the individual expresses a sense of companionship with the product.

### Implications for Future Wearable Technologies

While this study has identified key preferences, such as the size and weight of wrist-worn devices, from the perspectives of people with dementia, the wider aesthetic impacts of wearable technology remain underexplored. This includes how design choices across different body locations and device types (eg, VR-based technologies or crafted objects) may support therapeutic experiences, such as through artistic engagement [37] and memory recollection [82]. While some females with dementia express concerns about the lack of feminine appeal in current wearable designs, studies examining aesthetic rings and customized jewelry may reveal potential. This furthermore highlights the need for culturally appropriate design, as not all will identify with “feminine” aesthetics for inclusive design. In addition, soft, richly textured materials have shown promise in offering comfort and sensory reassurance, especially as memory loss advances and tactile needs increase [83]. Integrating these materials into long-term wearable designs may lessen the sense of detachment or discomfort often linked to medicalized or institutional-looking devices, instead fostering familiarity and emotional connection.

Together, these findings highlight the need for interdisciplinary collaboration between artists, designers, and technologists to develop devices that are not only functionally effective but also personally meaningful and aesthetically engaging, fostering long-term attachment and acceptance among users with dementia.

### The Need to Build a Positive Image of the Self

As shown in Figure 3, although current wearable technologies have increasingly focused on aesthetic enhancement and incorporated various design elements, such as the use of rich materials and multisensory features explored through innovative research on soft textiles and immersive VR tools, there remains limited understanding of how purely sensory experiences contribute to sustained emotional attachment or long-term adoption among individuals with dementia. A key next step in advancing these technologies is to deepen the understanding of how individuals interpret and form personal associations with such devices.

From a contrasting perspective, it is important to consider how people with dementia may perceive the idealized imagery of smart technologies and healthy aging narratives as alienating or even stigmatizing [43]. This highlights the need to evaluate the value and benefits of wearable technologies from the perspective of the wearers. To this end, co-design and participatory workshops may offer valuable opportunities to explore how people with dementia articulate their needs for monitoring sensors and technologies with varied purposes, their preferences regarding the form and feel of devices, and how these technologies can better support their lived experiences [33,84,85]. Rather than focusing solely on improving the feasibility of the products, developers and companies must consider where on the body these devices are preferred, how they should be integrated, and how much control individuals with dementia wish to retain, particularly concerning assistive functions, such as location tracking and close body monitoring.

### The Continuance of Self and Personalization

The disruption of selfhood is a longstanding concern in dementia care, with memory loss often seen as the key marker of identity decline. However, recent perspectives highlight that selfhood can persist through embodied actions, including everyday gestures, routines, and social behaviors, that continue even as memory fades [21]. These expressions, along with interactions with personal objects, reflect a form of identity beyond words.

In this context, the standardized, clinical aesthetics of wrist-worn wearables reveal a gap in supporting personal identity and emotional resonance. While crafted artifacts, such as bespoke jewelry, can foster attachment and convey empowering symbolism, most data-driven wearables prioritize function over individual expression [79]. This raises the need to reimagine wearables as more than passive sensing tools. Inspired by how VR enables users to construct personalized narratives and environments, wearable technologies could similarly support self-expression and identity continuity. Rather than static objects, they could become symbolic carriers of personal meaning, affirming agency and dignity through participatory, aesthetic engagement shaped from the moment of wearing.

### Strengths and Limitations

This review adopted a multidatabase search to capture a broad range of stakeholder perspectives on wearable technologies in dementia care. Two reviewers were involved to support a more flexible interpretation of the literature. However, as a critical interpretive synthesis, early-stage conceptual studies may have been excluded, potentially limiting insights into future design directions.

### Conclusion

This study conducted a critical interpretive synthesis, revealing a critical gap in the aesthetic design of wearable technologies for dementia care, which affects user acceptance and emotional engagement. Despite functional advances, adoption remains limited due to a lack of engagement in considering building personal meaning and sensory appeal. By analyzing 3 key themes influencing user-product interaction, this study proposes a new conceptual framework for understanding the role of aesthetics in dementia-focused wearable design. The findings point to the potential of aesthetic and inclusive design in shaping more engaging, acceptable, and human-centered wearable technologies for dementia care.

## Abbreviations

CIS: critical interpretive synthesis
PRISMA: Preferred Reporting Items for Systematic Reviews and Meta-Analyses
VR: virtual reality
